# Reaching back: the relative strength of the retroactive emotional attentional blink

**DOI:** 10.1038/srep43645

**Published:** 2017-03-03

**Authors:** Áine Ní Choisdealbha, Richard M. Piech, John K. Fuller, David H. Zald

**Affiliations:** 1 Economic and Social Research Institute, Whitaker Square, Sir John Rogerson’s Quay, Dublin, Ireland; 2 Department of Psychology, Anglia Ruskin University, East Road, Cambridge, Cambridgeshire, CB1 1PT, United Kingdom; 3 Department of Psychology, Vanderbilt University, 111 21st Avenue South, 301 Wilson Hall, Nashville, TN 37240, USA; 4 Department of Psychiatry and Behavioral Sciences, Vanderbilt University, 1601 23rd Avenue South Nashville, TN 37212, USA.

## Abstract

Visual stimuli with emotional content appearing in close temporal proximity either before or after a target stimulus can hinder conscious perceptual processing of the target via an emotional attentional blink (EAB). This occurs for targets that appear after the emotional stimulus (forward EAB) and for those appearing before the emotional stimulus (retroactive EAB). Additionally, the traditional attentional blink (AB) occurs because detection of any target hinders detection of a subsequent target. The present study investigated the relations between these different attentional processes. Rapid sequences of landscape images were presented to thirty-one male participants with occasional landscape targets (rotated images). For the forward EAB, emotional or neutral distractor images of people were presented before the target; for the retroactive EAB, such images were also targets and presented after the landscape target. In the latter case, this design allowed investigation of the AB as well. Erotic and gory images caused more EABs than neutral images, but there were no differential effects on the AB. This pattern is striking because while using different target categories (rotated landscapes, people) appears to have eliminated the AB, the retroactive EAB still occurred, offering additional evidence for the power of emotional stimuli over conscious attention.

Emotional visual stimuli gain access to conscious attention in a manner which is privileged over non-emotional stimuli, likely because of specialised neural mechanisms promoting detection of stimuli with ‘biological significance’^1^,^2^. There are many different contexts in which visual stimuli with affective content are perceived more quickly or hold attention more reliably than emotionally neutral stimuli. For example, targets that represent a threat, like snakes or angry faces, are detected more readily in spatial visual search than emotionally neutral targets^3^,^4^. Emotionally expressive faces dominate over neutral stimuli during binocular rivalry^5^ and fearful faces are more likely to be detected under some obscuring conditions than neutral faces^6^. Even detection of goal-relevant target stimuli may be slowed or impaired when emotional stimuli are present in the same visual scene^7^,^8^.

So great is the capacity of emotional stimuli to enter or hold awareness, that emotionally neutral stimuli that are being actively searched for can go completely undetected if they appear soon after a stimulus with affective content^9^. This phenomenon is called the emotional attentional blink (EAB) (or alternatively the emotional blink of attention or emotion-induced blindness). In this paradigm, task irrelevant distractors are presented in a rapid serial visual presentation (RSVP) stream, with the distractor appearing a few images prior to the target.

The EAB has similarities to the ‘standard’ attentional blink (AB)^10^,^11^, which occurs when an initial target stimulus (T1) hinders the ability to detect a second target stimulus (T2) occurring soon after the T1. Critically, in the AB, the effect occurs following a stimulus that the person is actively trying to detect. By contrast, the EAB occurs in response to a task irrelevant stimulus. Nevertheless, in general research on the EAB and AB has shown that these phenomena occur at similar lags relative to the distractor stimulus. Targets that appear 200 ms after emotional (non-target) stimuli in EAB studies, or nonemotional target (T1) stimuli in AB studies are usually not detected^9^,^12^,^13^. In each task, stimuli that appear 800 ms after the emotional distractor or T1 target can usually be detected^9^,^12^,^13^. These time-frames potentially suggest a similar time course of attentional allocation to emotional non-target and nonemotional target stimuli in EAB and AB tasks respectively. Additionally, robust privileging of emotional stimuli can also be seen in standard AB studies, where emotional face and word T2 stimuli are more likely to break through the AB than neutral T2 targets^14^,^15^. This may be due to the additive power of a stimulus that is simultaneously both emotional and a target, or due to a specific privileging of emotional stimuli.

Emotional stimuli can also impair target detection if they appear immediately *after* a target stimulus. Anderson^14^ reported an AB experiment in which accuracy at detecting T1 target words decreased if the T2 target appearing immediately after was a negatively-valenced, arousing word, relative to a neutral word. However, if T2 appeared as the second stimulus subsequent to T1 and was an emotional word, T1 accuracy increased relative to when T2 was neutral (although this pattern was inconsistent across different task variants and stimulus sets). A related study used EAB paradigm with emotional pictures appearing as distractors instead of targets and found similar results^16^. Their participants exhibited a retroactive or backward EAB, at a one-stimulus lag between T1 and a subsequent emotional distractor, and also showed an enhanced T1 detection at a two-stimulus lag between T1 and a subsequent emotional distractor.

Thus there appear to be at least three situations in which emotional stimuli preferentially capture attention relative to neutral stimuli in a RSVP – forward EAB, retroactive EAB, and detection of a T2 emotional stimulus. A critical research question is whether we can disentangle the mechanisms underlying emotional influences on attention in the AB and EAB. Specifically, do these phenomena reflect a single mechanism, or are they caused by distinct mechanisms? Neurobiological models of emotion and attention have evolved from an initial focus on a thalamic path to the amygdala as the primary mediator of the processing benefits given to emotional visual stimuli^17^,^18^, to broader perspectives on the roles of various cortical and subcortical networks in processing of visual stimuli with affective content^2^. This broader perspective provides an implicit or explicit acknowledgement of the possibility of different mechanisms underlying different kinds of attentional capture by, and attentional prioritising of, emotional stimuli. In partial support of this idea, in a recent fMRI study using emotional words Schwabe and colleagues^19^ found differential activity in the orbitofrontal and insular cortex vs. the amygdala depending upon whether emotional stimuli were causing an AB, or emotional T2 stimuli were breaking into awareness following detection of a T1 stimulus.

The present study investigated the relative strength of forward and retroactive EABs, and the ability of emotional stimuli to gain access to awareness during the refractory period of the standard AB. In order to try to measure a forward AB, a retroactive EAB, and the ability of emotional stimuli to break through an AB, we designed a novel dual-task RSVP paradigm in which subjects were asked to detect a T1 of a rotated landscape or architectural picture among a stream of upright landscape/architectural pictures, and then to detect a T2 target of persons who could be gory, erotic or neutral (clothed). This RSVP variant differs from a traditional AB paradigm in that the T1 and T2 are not drawn from the same class of stimuli. An immediate question arises as to whether an AB or a retroactive EAB will arise in such a design. If T1 and T2 detection rely on the same capacity-limited attentional resource, then both a traditional AB and a retroactive EAB should be observable. On the other hand, if detection and attentional resources can be committed in parallel, then neither phenomenon should be present. Thus an initial goal of this study was to determine whether AB and retroactive EABs were observable in a dual-task RSVP paradigm.

A second question addressed in this study is whether the effects of emotional stimuli from different affective categories produce similar effects in the forward and retroactive EABs or in AB breakthroughs. In our own work, erotic stimuli have demonstrated substantial and robust forward EABs. Indeed, these stimuli have produced larger EABs than gory or threatening stimuli^20^. Even in patients with PTSD, erotic stimuli produce larger EABs than combat related images^21^, but the effect of erotic vs. gory pictures in retroactive EAB’s is unknown.

Our final goal was to determine the congruence of emotion effects across the three types of demonstrated emotion effects in AB and EAB paradigms. We reasoned that if these three effects rely on the same mechanisms, then the relative ability of emotional stimuli to produce a forward EAB, a retroactive EAB and to break through the traditional AB should be similar. By contrast, if they rely on independent mechanisms they may be triggered by different stimulus characteristics. Other studies^16^,^22^,^23^ have used a correlational approach to explore the uniqueness of AB effects, albeit typically at the level of overall emotional categories, rather than individual stimuli. Kawahara and Kihara^23^ used this approach to determine whether the same mechanism underlies the AB (second target not identified due to detection of first target) and attentional capture (target not identified due to detection of salient distractors). Another study has investigated whether the forward EAB is correlated with the short-lag retroactive EAB^16^. Again in this case no correlation was found. In each of these studies, the authors suggest that separate mechanisms underlie the AB and the other attentional phenomena. This was examined at both the stimulus level (e.g., does a stimulus’s ability to be detected at T2 correlate with its ability to cause a forward or retroactive EAB?) and at the level of individual differences (e.g., does an individual’s sensitivity to forward or retroactive EAB effects correlate with their ability to detect emotional T2 stimuli?). If these stimulus level and individual differences level variables rely on a single mechanism, then they should be highly correlated. By contrast, if they reflect different mechanisms, they would be expected to show little correlation across stimuli or participants.

To address these questions, we asked participants to complete two RSVP paradigms. The first paradigm was similar to the design of Most and colleagues^9^, and tested for the forward EAB. Participants were asked to detect a rotated landscape or building among a stream of non-critical upright landscape or architectural images. One task-irrelevant critical distractor depicting people in a neutral, erotic or gory manner was presented in each trial with the distractor occurring either two stimuli (lag 2) or eight stimuli (lag 8) before the target. In a second block of trials, participants completed the dual task RSVP paradigm in which they had to detect both a T1 image (rotated landscape or building) and a T2 image containing a human (which could be gory, erotic or neutral), which allowed a determination of the extent to which the T2 stimulus could break through the AB and the extent to which the emotional T2 disrupted processing of the nonemotional T1 stimulus.

## Results

### Single Target Task

#### Forward EAB

**Figure 1:**
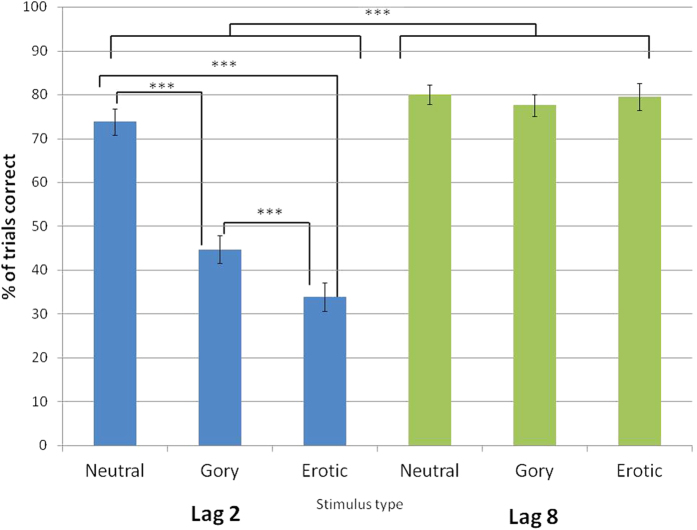
Number of targets identified at lag 2 and lag 8 following neutral, gory and erotic distractor stimuli in the single target task, indicative of forward EAB. Data expressed as mean ± SEM. ***p < 0.01.

The proportion of trials (out of a total 24) in which participants both detected and accurately reported the direction of the rotated landscape target when it appeared at a lag of two and eight stimuli following distractor images of different emotional categories is displayed in Fig. 1. Based on previous research showing that both emotional and non-affective blinks of attention substantially occur when the distractor occurs 2 stimuli prior to the target (lag 2^9^,^24^), we analyzed the impact of distractor category (neutral, erotic or gory) using repeated measures ANOVAs.

A significant main effect of distractor category was found, F (2, 52) = 58.26, *p* < 0.001, *η*^*2*^ = 0.69, in addition to a main effect of lag, F (1, 26) = 186.53, *p* < 0.001, *η*^*2*^ = 0.88, and an interaction between distractor category and lag, F (2, 52) = 75.41, *p* < 0.001, *η*^*2*^ = 0.74. Planned comparisons using Fisher’s least significant difference (LSD) method revealed significant differences between each stimulus class at lag 2 but not lag 8. At lag 2, attentional blinks following erotic stimuli occurred significantly more often than after gory pictures (*p* < 0.001, mean difference in detection 10.8% (SE 2.35%), and following gory (*p* < 0.001, mean difference 29.17% (2.61%)) and erotic (*p* < 0.001, mean difference 39.97% (3.07%)) stimuli more often than after neutral ones. In contrast, performance at lag 8 was consistently high. There were no significant differences in stimulus detection following the different distractor categories at this lag (all p > 0.1). At lag 8, across all categories, a mean of 79.12% of stimuli were detected (SE = 1.43).

### Dual Target Task

#### Retroactive EAB

**Figure 2:**
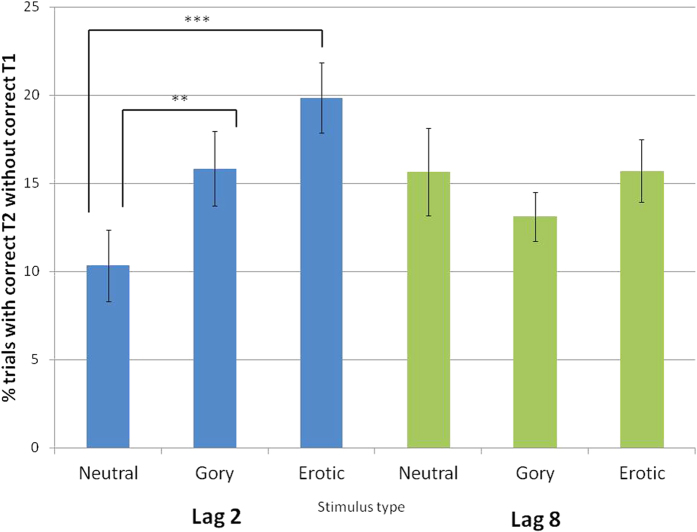
Proportion of trials with correctly-identified neutral, gory and erotic T2s without identification of rotated landscape T1 in the dual target task, indicative of retroactive emotional attentional blink. Data expressed as mean ± SEM. ***p < 0.01, **p < 0.05.

Trials in which the T2 target stimulus containing a person was identified correctly but the rotated landscape T1 target stimulus was not were classified as showing a retroactive EBA. This is represented in Fig. 2 as the proportion of trials with a correctly identified T2 that did not have a correctly identified T1. A 2-by-2 lag-by-stimulus type repeated measures ANOVA showed a main effect of T2 stimulus category on the ability to detect the T1 rotated landscape, F (2, 52) = 4.91, *p* = 0.011, *η*^*2*^ = 0.16. There was no main effect of lag, F (1, 26) = 0.03, *p* = 0.6, *η*^*2*^ < 0.01 but a significant interaction between lag and T2 stimulus category was found, F (2, 52) = 5.33, *p* = 0.008, *η*^*2*^ = 0.17. Planned comparisons using Fisher’s LSD revealed that retroactive blinks occurred more often for both gory (*p* = 0.007, mean difference 5.5% (SE = 1.88%)) and erotic (*p* = 0.0003, mean difference 9.51% (2.25%)) T2 targets than for neutral T2 targets at lag minus-2. Additionally, at this lag there were more trials in which a T1 target appearing before an identified erotic stimulus was not identified than trials in which a T1 target appearing before an identified gory stimulus was not identified (p = 0.049, mean difference 4.01% (2.34%)). As expected, at lag minus-8, no effects of T2 category were found (neutral vs. gory *p* = 0.828, neutral vs. erotic *p* = 0.489, gory vs. erotic *p* = 0.062). At this lag, across all categories, a mean of 14.83% of rotated T1 targets were not detected when a T2 person stimulus was (SE = 1.07%). Thus, the dual-target paradigm demonstrates the presence of a retroactive EAB at lag minus-2 that is sensitive to the emotional content of stimuli, with erotic images producing the largest retroactive EAB, similar to erotica’s greater effect in the forward EAB paradigm. Results indicate no difference in the frequency of T2 detection without T1 across both lags.

#### Lack of an Attentional Blink Limits the Ability to Observe an AB Breakthrough

**Figure 3:**
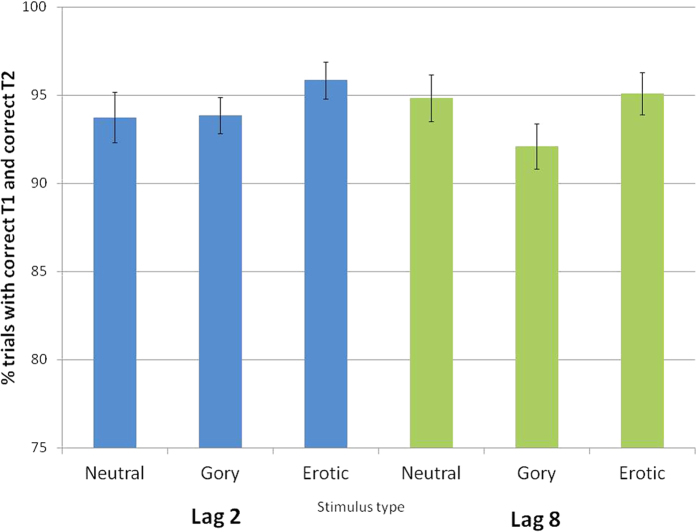
Proportion of T2 targets identified following successful T1 identification in the dual target task, indicative of AB breakthrough (note general lack of AB). Data expressed as mean ± SEM.

To examine the presence of an AB caused by detection of the T1 rotated target, we identified all trials in which a rotated landscape T1 was correctly detected (all necessarily different trials from those used in the retroactive EBA analysis), and then determined whether the T2 was correctly found. Strikingly, there was little evidence of a typical forward AB, in that T2 stimuli were detected at high levels despite appearing after a successfully detected T1. The percentage of T2 stimuli that were successfully detected for all stimulus categories at both lags ranged from 91.4% to 95.5% (see Fig. 3). The high success rate in identifying even neutral T2s limits the ability to observe even a small AB breakthrough effect. We, nevertheless, performed planned analyses to test for differential effects based on T2 categories. A repeated measures ANOVA revealed a marginal effect of T2 stimulus category on breakthrough, F (2, 52) = 2.97, *p* = 0.06, *η*^*2*^ = 0.1; with no main effect of lag, F (1, 26) = 0.49, *p* = 0.49, *η*^*2*^ = 0.02; and no significant interaction between lag and T2 stimulus category, (2, 52) = 1.16, *p* = 0.32, *η*^*2*^ = 0.04. Across all conditions, 94.26% of person-containing stimuli were identified (SE = 0.48%).

#### Picture-based analysis

In addition to looking at participant performance at detecting targets, we also investigated the performance of each stimulus in eliciting the different attentional processes. It was assumed that if the retroactive EAB relies on the same processes as the forward EAB, each stimulus would elicit these processes in similar proportions. As a means of determining this, the number of times emotional stimuli caused a forward (single target task/task 1) or retroactive (dual target task/task 2) EAB or a breakthrough of the traditional AB across all subjects was calculated and submitted to correlation analysis. (We note that we include analysis of the AB breakthrough for thoroughness as these were planned analyses. However, the lack of evidence for an AB in the dual task design limits the interpretation of these correlations.) The picture-based data were not normally distributed (all Shapiro-Wilk tests *p* < 0.05). Consequently Spearman’s rho was used in the correlation analyses. Alpha levels for this and the individual differences correlation below were set at α = 0.05 as it was hypothesised that a positive correlation would be found between proportion/number of blinks in all cases.

Descriptive data shows that neutral stimuli caused forward blinks of attention on 17.13% of the lag 2 trials in which they were present (SE = 2.45%). For gory stimuli, this was 35.03% (3.28%) and for erotic stimuli, it was 39.97% of trials (2.88%). The respective numbers for retroactive blinks per stimulus at lag 2 were neutral 12.19% (1.72%), gory 15.43% (2.43%); erotic 20.06% (3.15%) and for breaking through the attentional blink they were neutral 85.34% (2.02%); gory 80.4% (2.51%); and erotic 77.47% (3.79%). The number of times an image caused a forward EAB when it appeared at a lag of two stimuli prior to the target in the single target task was not correlated with the number of times it caused a retroactive EAB when it appeared as a T2 target at a lag of 2 stimuli following the landscape target in the dual target task, *r* = 0.063, *p* = 0.598. There was no significant correlation between the number of times a stimulus caused a forward EAB and the number of times it broke through the traditional AB either (lag 2, *r* = −0.211, *p* = 0.075). The correlations between the forward EAB, the retroactive EBA and attentional breakthroughs broken down into stimulus category are listed in Table 1.

#### Individual differences analyses

In order to further investigate the relationship between the studied attentional processes, correlation analyses were conducted to determine if those participants who were particularly prone to forward EABs were also prone to retroactive EABs or to AB breakthrough by affective stimuli. The Pearson product moment correlation between the proportion of trials in which a participant experienced a forward EAB and the proportion of trials in which they experienced a retroactive EAB was not significant at lag 2 (*r* = −0.092, *p* = 0.649) (see Table 2 below for stimulus category correlations). For the correlation between the proportion of trials in which a participant experienced the forward EAB and the proportion of trials in which they experienced a stimulus breaking through the AB window, no significant effects were found, *r* = 0.132, *p* = 0.513 (although interpretation of this result is limited by the lack of evidence for an AB in the dual task design).

## Discussion

Several findings emerge from the above studies of the EAB. The data from the first task reaffirm the robust forward EAB phenomenon and confirm the particularly powerful nature of erotica in eliciting an EAB^20^,^21^. The more novel findings relate to the dual target RSVP task. We found that detection of a non-affective T1 target appearing two stimuli prior to a second, person-containing target is impaired more frequently when the second target is gory or erotic than when it is affectively neutral. However, this task did not produce a “traditional” AB. Even neutral images were detected at a high level during the traditional AB window.

The finding of a retroactive EAB at a lag of two stimuli between the emotional and the non-emotional targets (lag minus-2) differs from the results of previous research by Anderson^14^ and Most and Jungé^16^. These authors found that emotional stimuli presented immediately subsequent to target stimuli (lag minus-1) impaired detection of the target stimuli, but that emotional stimuli presented two stimuli following non-emotional targets (lag minus-2) facilitated target detection. The reason why we find impairment where these other studies find facilitation may arise from differences in task design. In the work of Most and Jungé^16^, the emotional stimuli were task-irrelevant distractors. In our work, they were targets. Anderson^14^ presented the emotional stimuli as T2 targets, but they shared colour features with the non-emotional T1 target words. By contrast, in our work, participants had two tasks and consequently had to be vigilant for two types of target; they had to detect the rotated landscape target (and identify the rotation direction), and detect and report the state (clothed, naked or injured) of the person in the other target. The present data confirm that retroactive EAB effects exist, and indicate that at least under certain conditions they can extend to a longer lag than previously shown. The fact that at a 200 ms lag emotional stimuli can either enhance (as per other research) or impair (as per this research) T1 detection relative to neutral stimuli suggests that diverse processes can occur when an emotional stimulus appears while a representation of a preceding target is active, depending on task demands. Given the above-listed multiple differences between this task and others’ work^14^,^16^, the specific factors leading to differences in the strength and timing of a retroactive EAB remain to be determined. It is noted that the retroactive EAB was seen in a small number of trials, relative to the forward EAB. Other retroactive EAB research using emotional distractors at a shorter, 1-stimulus lag reports identification of targets at a similar rate (over 80% of trials).

In the dual-target task, it is noteworthy that no “traditional” attentional blink is seen, suggesting that T1 had little impact on T2 processing. The lack of AB effect prevents direct comparison of the processes involved in and manifestation of AB breakthrough versus the retroactive emotional blink in this study. There are many possible reasons for the asymmetry of T1 (AB) and T2 (retroactive EAB) effects in the dual-task RSVP task, reflecting the fact that T1 and T2 stimuli were of different types. In the dual-task RSVP stream, T1 stimuli were similar to the distractor images as both were comprised of landscape and architectural images, which only differed from each other in whether they were upright or rotated. In contrast, the T2 came from an entirely different class of images, and there was never more than one image of a person shown per trial. This may have allowed for some degree of parallel processing. Executive control of attention can allow more than one attentional focus to be maintained in this context^25^. Evidence for such parallel processing in an EAB design has been reported by Most and Wang^26^, who exposed participants to two spatially distinct RSVPs at the same time, and found that emotional stimuli only interfered with targets in the same RSVP as the emotional distractor, not affecting the target in the spatially distinct RSVP. The categorical difference between T1 and T2 stimuli, and categorical similarity between T1 and distractor stimuli, may also have led to a difference in the degree of discrimination or elaboration necessary to identify targets that made the T1 target more vulnerable to competition than the T2 stimulus, or that simply allowed T2 to “pop-out” due to its differences from surrounding stimuli.

Another reason for the asymmetry may have been that the questions about the T2 targets required high-level (clothing) details about these stimuli and thus participants were more vigilant for T2 than T1 targets. The emotional T2 stimuli shared low-level characteristics with the rotated T1 stimuli (i.e. bodies depicted being strongly in the horizontal plane) whereas the neutral T2 stimuli were more vertical and thus more similar to the distractors. Thus, if a T1 identification lapse occurred, participant identification of an emotional (gory or erotic) T2 would be more likely than identification of a neutral T2 because the emotional T2s shared some T1 target criteria (a strong horizontal feature). A final possible explanation for the asymmetry of retroactive EABs and traditional ABs is that the T2 targets bore strong biological significance^27^ because they contained people, and thus were generically privileged in their detection in spite of a previously-detected neutral T1 target. Although performance in the dual target task was high, significant emotion-based differences did emerge among those trials in which only the second target was detected. Emotional stimuli can, at least in a minority of trials, still capture resources that would have been allocated to elaboration of the earlier T1.

The differential effects of stimulus type accord with previous studies that have found that the particular emotional category of a stimulus affects its ability to capture or hold attention^15^,^28^. There is precedent for the particular ability of erotic stimuli to hold visual attention better than negatively valenced stimuli, as it was found that sexual words caused an EAB in a word-based RSVP paradigm, whereas non-sexual positive, negative, anxiety-related and threat-related words did not^29^. The present study found that not only did erotic stimuli cause forward EAB and retroactive EAB at two-stimulus lags, they did so significantly more often than gory images (which also caused EABs). We note that the patterns of effects were similar for both EABs although not directly comparable given the fact that the stimuli were task-irrelevant distractors in the single target task and targets in the dual target task. This shift from task-relevance to task-irrelevance may lead to processing of the target emotional stimuli in the dual-target task that would be different if the stimuli were always task-relevant. We also note that there were fewer trials included in the retroactive EAB analysis due to the relatively low occurrence of the retroactive EAB (less than one-fifth of dual target task trials). Although the present results appear to suggest that stimulus valence affects the forward EAB but not the retroactive EAB, it must be noted that ratings by an independent sample of subjects, the erotic stimuli were rated as being more arousing than the gory stimuli and consequently the relative effects of arousal and valence cannot be determined. The number of trials in which an emotional second target was identified but a first target landscape was not were similar at lag-minus-2 and lag-minus-8 (that is, there was no effect of lag in relation to the retroactive blink).

The other research question addressed in this study – that of whether the forward and backward EABs rely on the same mechanism – was investigated by correlating the number of times each stimulus caused a forward blink (across subjects) with the number of times it caused a backward blink. There was no correlation between the two blinks, at either lag or for any of the stimulus categories. Because each stimulus is a static entity which presumably maintained the same arousal value^30^, valence^28^ and biological/social significance^27^ throughout the study (other than possible changes in value due to habituation), the lack of correlation suggests that the stimulus properties that make a given stimulus most susceptible to a forward and (dual-task) retroactive EAB are different. This may also suggest that there are different mechanisms subserving the forward and retroactive EABs. Such a conclusion is supported by other findings^19^ that show that the enhanced processing of emotional T1 stimuli is associated with a different neural pathway than the processing of emotional T2 stimuli. The question of why stimuli that hold attention strongly enough to cause subsequent target misses are not identical to those that are most likely to capture attention away from and interfere with the processing of previously attended stimuli requires future investigation. It is possible that features like arousal value and biological significance play different roles in these processes. However, we must acknowledge that methodologically the current study may not have provided an ideal test for this. With retroactive blinks observed on no more than 20% of trials for a given category, the psychometric properties of the task may have limited the ability to observe associations. The correlation between forward EABs and AB breakthroughs per image was also analysed for completeness but given the lack of AB effect, no overall conclusions can be drawn.

Further support for the idea that the forward and retroactive EAB are not based on identical mechanisms comes from the lack of a correlation between susceptibility to the forward EAB and retroactive EAB. If identical mechanisms were employed for each of these processes, it would be predicted that those who are most susceptible to one form of EAB (e.g. participants high in trait harm avoidance^9^) would be most susceptible to the other form. This conclusion is limited by the fact that it is based on a null result, but seems reasonable given the limited data available at present and the conclusions of other studies using a similar approach^16^,^23^.

The results of these correlation analyses align with existing research suggesting that the mechanisms by which attention is captured or held by salient stimuli differ depending on whether the stimulus is a target or distractor^23^ and whether it appears before or after a target stimulus^16^. The present work nonetheless provides evidence for a retroactive EAB in a dual-target task that, like the forward EAB, is differentially induced by erotic and gory stimuli. Although relatively weaker than the forward EAB, the dual target task’s retroactive EAB is stronger for erotic than gory stimuli – just like the forward EAB. This suggests that the capture and holding of attention by emotional stimuli depends on stimulus features, potentially including arousal value or valence – regardless of whether the same or different mechanisms underlie each form of EAB studied here.

## Methods

### Participants

Given the particularly robust affective responses typically produced when males view erotic pictures^20^,^31^, only males participated in the present study. They had an age range of 18 to 25 years and all were students at Vanderbilt University who participated in return for course credit. The study was approved by the Vanderbilt Institutional Review Board and carried out in accordance with their guidelines. All participants gave written informed consent to participate. Prior to signing up for the study, participants were told that the study was specifically recruiting males who were sexually attracted to women. During the consent process they were additionally told that if they were not sexually attracted to women, they could withdraw without explanation and that no record would be kept if they withdrew prior to completion of written consent.

Data from 31 participants were collected, but data from only 27 of these were included in the analysis. Two participants were excluded because they participated in only one of the two tasks, and two were excluded because they identified two times the standard deviation fewer targets than the mean for the participant group in either of the two tasks.

### Stimuli

Stimuli were colour photographs 9.5 cm wide by 7.5 cm tall, each adjusted to have the same luminosity. The target stimuli for both tasks were 192 images of landscapes, rotated 90 degrees clockwise or 90 degrees anti-clockwise. Non-critical distractors were 252 images of landscapes presented in an upright orientation. Stimuli used as critical distractors in the single target EAB task (Task 1) and T2 targets in the dual-target (Task 2) came from multiple sources. Those with neutral affective content came from the International Affective Picture System (IAPS)^32^. There were 72 of these in total and they all depicted fully-clothed women set against neutral backgrounds, and each image was rated within the IAPS as having a neutral valence rating and a low arousal rating. Supplemental gory and erotic stimuli were found via internet image searches. There were 60 gory stimuli, each depicting mutilated, injured or lacerated human figures. There were also 60 erotic stimuli, all of which depicted fully nude female figures. These were rated for arousal and valence by a separate group of young (18- to 25-year-old) male participants. Gory stimuli were given a significantly lower mean valence rating and erotic stimuli were given a significantly higher mean valence rating than the neutral IAPS stimuli used (identical to those used in the present experiment). The specific figures are as follows, all stimuli rated from −100 (most negative valence) to 100 (most positive valence); neutral M = 7.8 (SD = 10); gory −61.1 (16.4); erotic 45 (15.8). Both erotic and gory stimuli had significantly higher arousal ratings than neutral stimuli, with erotic stimuli also being significantly more arousing than gory stimuli. Arousal was rated on a scale from 0 to 100 (lowest to highest arousal); neutral 23.6 (17.5); gory 58.4 (15.5); erotic 65 (16). Stimuli were presented on a 17-inch CRT monitor using E-Prime (Psychology Software Tools, Pittsburgh, PA).

### Procedure

Both the single target and dual target RSVP tasks followed a similar structure. In each trial, 17 images were presented for 100 ms each. Fifteen of these images were non-critical distractors (that is, upright landscapes). The other two were one each of a rotated landscape target and a stimulus that contained a person. Both tasks contained 6 blocks of 32 trials, making for 192 trials in total per task. In each task, there were 96 “lag 2” or “lag minus-2” and 96 “lag 8” or “lag minus-8” trials.

**Figure 4:**
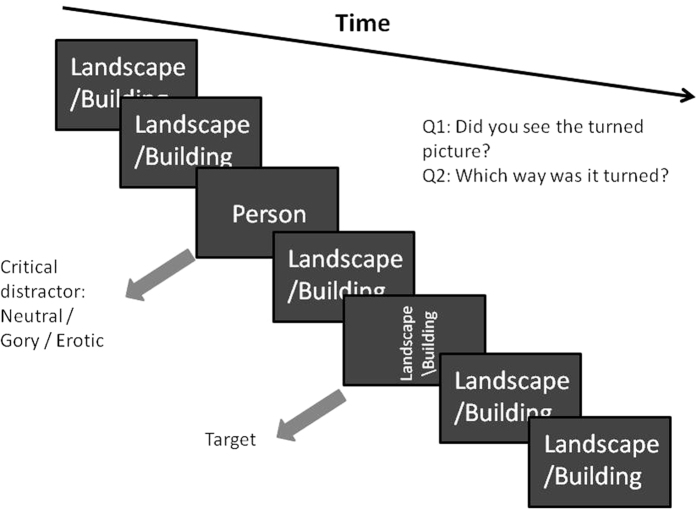
Schematic depiction of trial structure in the first (single target) task.

In each trial of the single target task (Fig. 4), the stimulus with a person served as task-irrelevant critical distractor. The critical distractor occurred on the 4^th^, 6^th^ or 8^th^ stimulus in the RSVP stream. The rotated landscape target appeared as the 2^nd^ (lag 2) or 8^th^ (lag 8) stimulus subsequent to the critical distractor. After each trial, participants were asked, “Did you see a rotated landscape?” and had to respond with a keypress indicating “yes” or “no”. If they responded with a “yes” keypress, they were then asked the second question, “Which way was it rotated?” and had to respond with a keypress indicating “left” or “right”. The next trial began immediately following their answer. Trials in which a person affirmed that they had seen a landscape but responded with the wrong orientation were classified as trials in which a blink had occurred.

**Figure 5:**
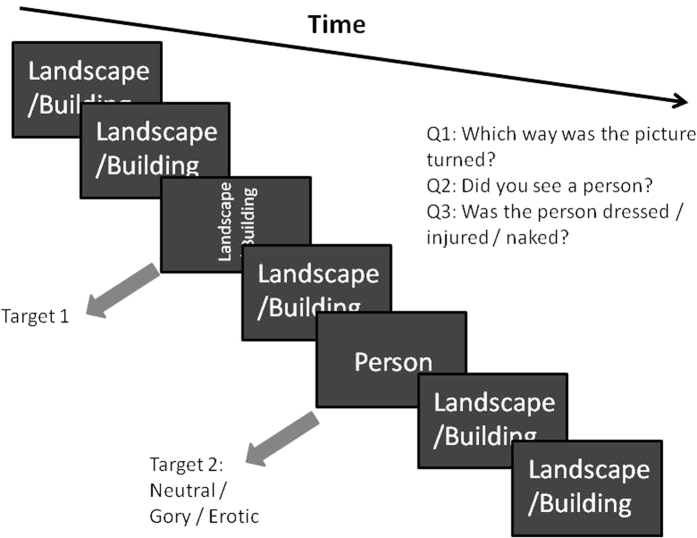
Schematic depiction of trial structure in the second (dual target) task.

In the second task, participants had to identify two targets (Fig. 5). Like in the first task, one of these critical stimuli appeared as the 4^th^, 6^th^ or 8^th^ stimulus in the RSVP stream and the other appeared 2 (lag minus-2) or 8 (lag minus-8) stimuli subsequent to it. The same stimuli as used in the first task were used in this second task but due to the short presentation time of stimuli, the large number of them, and a break given between the tasks, recognition or repeat exposure are unlikely to have caused significant problems with the data. In this dual target task, the rotated landscape stimulus appeared as the first target (T1) and the stimulus displaying a person appeared as the second target (T2). After each trial the following three questions were asked in sequence, with the third question only appearing if the answer to the second question was “yes”: 1) Which way was the rotated landscape turned?” (keypress “left” or “right”); 2) “Did you see a person?” (keypress “yes” or “no”); 3) “Was the person a) clothed b) naked c) injured?” (keypress “clothed”, “naked” or “injured”). Again, trials in which the participant incorrectly identified the orientation of the landscape or the features of the person-containing stimulus were classified as trials in which a target was missed.

## Additional Information

**How to cite this article**: Ní Choisdealbha, A. *et al*. Reaching back: the relative strength of the retroactive emotional attentional blink. *Sci. Rep.* **7**, 43645; doi: 10.1038/srep43645 (2017).

**Publisher's note:** Springer Nature remains neutral with regard to jurisdictional claims in published maps and institutional affiliations.

## Figures and Tables

**Table 1:** Picture-based correlations between blinks and breakthroughs across stimulus category at lag 2.

Category	Measure	Neutral	Gory	Erotic
Forward vs. retroactive blink	*r*	−0.283	−0.128	0.158
	*p*	0.181	0.551	0.461
Forward blink vs. AB breakthrough	*r*	0.035	−0.108	−0.165
	*p*	0.871	0.614	0.441

All analyses Spearman’s rho.

**Table 2:** Correlations between the number of times each participant experienced forward and backward EBAs (lag 2).

Category	Measure	Neutral	Gory	Erotic
Forward vs. backward blink	*r*	−0.226	−0.076	0.037
	*p*	0.258	0.705	0.855
Forward blink vs. AB breakthrough	*r*	0.262	0.089	0.039
	*p*	0.187	0.658	0.846

All analyses Pearson’s product moment correlation.
